# P-1164. Targeting Antibiotic-Resistant and Biofilm-Forming Staphylococcus aureus with BX211 Phage Therapy

**DOI:** 10.1093/ofid/ofaf695.1357

**Published:** 2026-01-11

**Authors:** Ariel Cohen, Benjamin A Lipsky, Siblian Boston, Christine Orlando, Mike Sowers, Joe Fackler, Rima Sandhu, Aravinda Vadlamudi, Rob cohen, Anantha Makineni, Edward Fang, Robert Hopkins, Jagoda Jablonska, David Zlotin, Ron Mordoch, Edith Kario, Jenia Gold, Maya Olshina, Shiran Hovav, Myriam Golembo, Nitsan Halevy, Hila Sberro Livnat, Merav Bassan

**Affiliations:** BiomX, Ness Ziona, HaMerkaz, Israel; University of Washington, Seattle, Washington; BiomX Inc, Gaithersburg, Maryland; BiomX Inc, Gaithersburg, Maryland; BiomX Inc, Gaithersburg, Maryland; BiomX Inc, Gaithersburg, Maryland; BiomX Inc, Gaithersburg, Maryland; BiomX Inc, Gaithersburg, Maryland; BiomX Inc, Gaithersburg, Maryland; BiomX Inc, Gaithersburg, Maryland; BiomX Inc, Gaithersburg, Maryland; BiomX Inc, Gaithersburg, Maryland; BiomX, Ltd, Ness Ziona, HaMerkaz, Israel; BiomX Ltd, Ness Ziona, HaMerkaz, Israel; BiomX Ltd, Ness Ziona, HaMerkaz, Israel; BiomX, Ltd, Ness Ziona, HaMerkaz, Israel; BiomX, Ltd, Ness Ziona, HaMerkaz, Israel; BiomX Ltd, Ness Ziona, HaMerkaz, Israel; BiomX Ltd, Ness Ziona, HaMerkaz, Israel; BiomX, Ltd, Ness Ziona, HaMerkaz, Israel; BiomX Ltd, Ness Ziona, HaMerkaz, Israel; BiomX, Ltd, Ness Ziona, HaMerkaz, Israel; MB, Ness Zionna, HaMerkaz, Israel

## Abstract

**Background:**

Bacteriophages (phages) are viruses that selectively infect and lyse bacteria. Unlike antibiotics, phages are highly specific to their targets, can replicate at infection sites, and may degrade or penetrate biofilms-an advantage in antibiotic-resistant or biofilm-associated infections. Their specificity also minimizes impact on the surrounding microbiome. Diabetic foot osteomyelitis (DFO), often caused by *Staphylococcus aureus* (S. aureus), is a chronic infection frequently associated to biofilm formation and prolonged antibiotic use. Phage therapy may complement standard treatment by providing targeted antibacterial activity, including against pathogens that are antibiotic-resistant or embedded within biofilms.

**Methods:**

In a randomized, double-blind, placebo-controlled Phase 2b study, patients with culture-confirmed S. aureus-positive DFO received phage therapy plus standard antibiotics. Phages were selected per patient from a GMP-manufactured bank based on lytic activity against each baseline isolate, ensuring individualized coverage and enabling longitudinal susceptibility monitoring.

**Results:**

About 90% of S. aureus isolates were susceptible to at least one phage. Notably, this broad coverage was achieved with a relatively small number of phages, supporting the feasibility of formulating an efficient cocktail for future studies. No phage resistance emerged during treatment. Many isolates showed high in vitro biofilm production; combined with positive clinical signals, this suggests phages may have contributed to efficacy via biofilm degradation. The most common sequence type was ST8, including USA300 MRSA, a major cause of skin and soft tissue infections. Phages showed strong activity against these strains, highlighting the clinical relevance of this approach.

**Conclusion:**

This trial demonstrates phage therapy's feasibility and clinical integration for DFO, with high strain match rates, targeted coverage of virulent and resistant S. aureus, and relevance to biofilm-associated infection biology.

**Disclosures:**

Ariel Cohen, PhD, BiomX Ltd: Employee Benjamin A. Lipsky, MD, FACP, FIDSA, FRCP, FRCPS, BiomX: Advisor/Consultant|BiomX: Honoraria Siblian Boston, MSc, BiomX Inc: Employee Christine Orlando, MSc, BiomX Inc: Employee Mike Sowers, BS, BiomX Inc: Employee Joe Fackler, PhD, BiomX Inc: Employee Rima Sandhu, PhD, BiomX Inc: Employee Aravinda Vadlamudi, MSc, BiomX Inc: Employee Rob cohen, MSc, MBA, BiomX Inc: Employee Anantha Makineni, PhD, BiomX Inc: Employee Robert Hopkins, MD, TolkaAI: CMO|TolkaAI: Stocks/Bonds (Private Company) Jagoda Jablonska, PhD, BiomX Ltd: Employee David Zlotin, Msc, BiomX Ltd: Employee Ron Mordoch, MSc, BiomX Ltd: Employee Jenia Gold, M.Sc, BiomX Ltd: Employee Maya Olshina, PhD, BiomX Ltd: Employee Shiran Hovav, PhD, BiomX Ltd: Employee Myriam Golembo, PhD, BiomX Ltd: Employee Nitsan Halevy, MD, BiomX Ltd: Advisor/Consultant Hila Sberro Livnat, PhD, BiomX Ltd: Employee Merav Bassan, PhD, BiomX: Employee|BiomX: Stocks/Bonds (Public Company)

